# Evaluating cancer genetic services in a safety net system: overcoming barriers for a lasting impact beyond the CHARM research project

**DOI:** 10.1007/s12687-023-00647-x

**Published:** 2023-05-01

**Authors:** Sonia Okuyama, Larissa L. White, Katherine P. Anderson, Elizabeth Medina, Sonia Deutsch, Chelese Ransom, Paige Jackson, Tia L. Kauffman, Kathleen F. Mittendorf, Michael C. Leo, Joanna E. Bulkley, Benjamin S. Wilfond, Katrina AB Goddard, Heather Spencer Feigelson

**Affiliations:** 1 Division of Oncology, Denver Health and Hospital Authority, Denver, CO USA; 2 Institute for Health Research, Kaiser Permanente, Aurora, CO USA; 3 Ambulatory Care Services, Denver Health and Hospital Authority, Denver, CO USA; 4 Denver Health and Hospital Authority, Denver, CO USA; 5 Kaiser Permanente Center for Health Research, Portland, OR USA; 6 Vanderbilt University Medical Center, Nashville, TN USA; 7 Department of Translational and Applied Genomics, Kaiser Permanente Center for Health Research, Portland, OR USA; 8 Treuman Katz Center for Pediatric Bioethics, Seattle Children’s Research Institute and Hospital, Seattle, WA USA; 9 Department of Pediatrics, University of Washington School of Medicine, Seattle, WA USA; 10 Division of Cancer Control and Population Sciences, National Cancer Institute, Bethesda, MD USA

**Keywords:** Genetic services, Barriers, Research implementation, Research collaboration

## Abstract

Underserved patients face substantial barriers to receiving cancer genetic services. The Cancer Health Assessments Reaching Many (CHARM) study evaluated ways to increase access to genetic testing for individuals in underserved populations at risk for hereditary cancer syndromes (HCS). Here, we report the successful implementation of CHARM in a low-resource environment and the development of sustainable processes to continue genetic risk assessment in this setting. The research team involved key clinical personnel and patient advisors at Denver Health to provide input on study methods and materials. Through iterative and collaborative stakeholder engagement, the team identified barriers and developed solutions that would both facilitate participation in CHARM and be feasible to implement and sustain long term in clinical care. With a focus on infrastructure building, educational modules were developed to increase awareness among referring providers, and standard methods of identifying and managing HCS patients were implemented in the electronic medical record. Three hundred sixty-four DH patients successfully completed the risk assessment tool within the study, and we observed a sustained increase in referrals to genetics for HCS (from 179 in 2017 to 427 in 2021 post-intervention). Implementation of the CHARM study at a low-resourced safety net health system resulted in sustainable improvements in access to cancer genetic risk assessment and services that continue even after the study ended.

**Trial registration** NCT03426878

## Background

Underserved and marginalized populations experience substantial barriers to receiving cancer genetic services and care (McCarthy et al. 2016, Shields, Burke and Levy 2008, Cragun et al. 2017, Meyer et al. 2010, Smith et al. 2016, Delikurt et al. 2015, Flynn et al. 2010, Murff, Byrne and Syngal 2004, Nippert et al. 2011, Plat et al. 2009, Sin et al. 2018, Wood et al. 2014). Research efforts seek to identify and overcome these barriers, but improvements in the process of care that take place as part of a research program often are not sustainable when the study ends. This problem is more pronounced in low-income, low-education, limited English proficiency, and minority populations (Koh et al. 2010, Roberts et al. 2019, Rana et al. 2018). Safety net medical care settings may find it difficult to translate these interventions into sustained practice improvements (Srinivasan et al. 2021).

The Cancer Health Assessments Reaching Many (CHARM) study evaluated ways to increase access to evidence-based genetic testing for hereditary cancer in low-income and low-literacy populations in diverse primary care settings (Mittendorf et al. 2021, Amendola et al. 2018). CHARM recruited adults who were at increased risk for hereditary cancer syndromes (HCS) based on family history and could benefit from genetic testing to inform the need for earlier and more frequent cancer surveillance or consideration of interventions for cancer risk reduction. In this paper, we describe how the implementation of the CHARM study at a low-resourced health system resulted in sustainable changes that allowed the work of genetic cancer risk assessment to continue, even after the study ended.

## Methods

### Setting

**Fig. 1:**
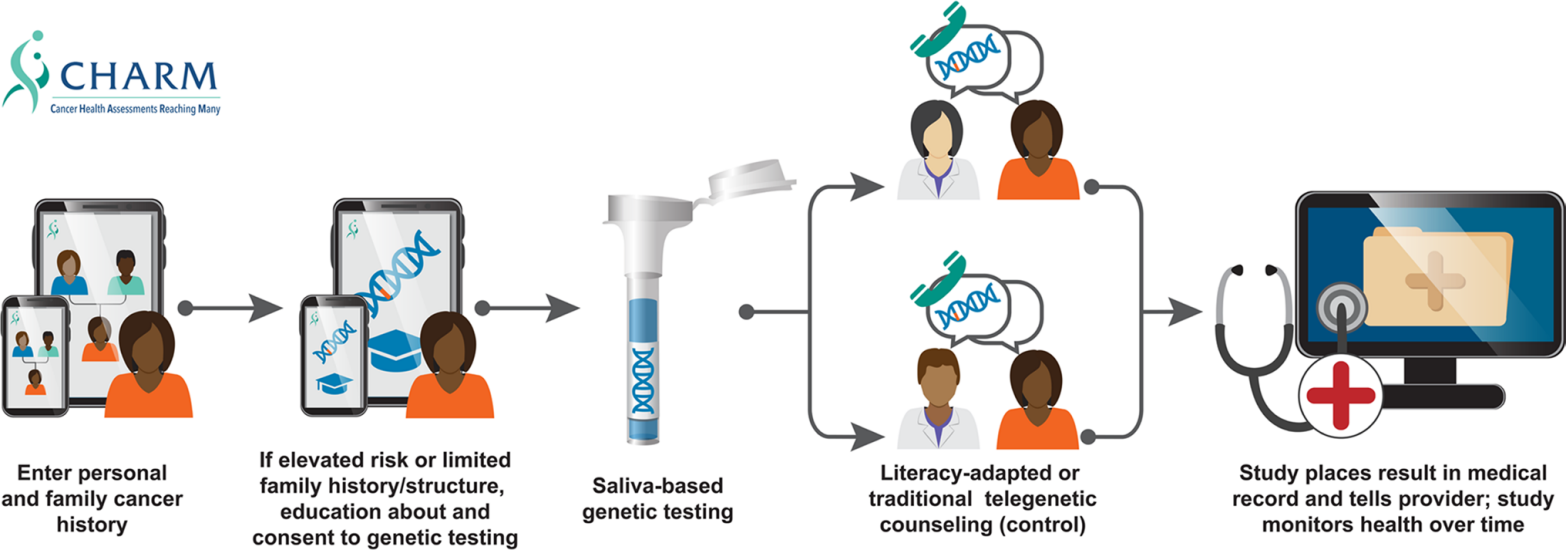
Description of CHARM study

The CHARM study has been described elsewhere (Mittendorf et al. 2021, Mittendorf et al. 2022b). Briefly, CHARM investigators developed a novel, literacy-adapted electronic patient-facing family history risk assessment tool in Spanish and English using two clinically validated clinician-facing modules (B-RST™3.0 and PREMM^5^) that patients would complete outside of a clinical encounter on a portable electronic device or computer. If a patient screened at high risk for HCS, or lacked information about their family history, they received education about genetic testing and were invited to participate in the study. Risk assessment, pre-test genetic counseling, and informed consent for the study were all incorporated into this interactive tool, without the need of any clinician’s involvement. Study participants provided saliva for clinical genomic sequencing and were randomized to receive two styles of genetic counseling where their results from the genetic testing were disclosed. The participant’s clinical team also received results of genetic testing and counseling recommendations (Fig. 1). Recruitment for CHARM occurred at Kaiser Permanente Northwest (KPNW) in Oregon and Southwest Washington and Denver Health (DH) in Denver, Colorado, from August 2018 to March 2020. Patients were eligible to participate if they were between the ages of 18 and 49 years and spoke English or Spanish. Here, we limit our discussion to implementation at DH given the unique challenges of conducting research in a low-resource environment.

DH is an integrated health care system with a robust primary care network that, at the time of the study, consisted of nine Federally Qualified Health Centers (FQHCs) located in Denver’s poorest neighborhoods and 18 school-based clinics, providing primary care to approximately 175,000 patients (about one-third of Denver County residents). As of 2019, 75% of DH patients lived in poverty, defined as having an income of less than 200% of the federal poverty level. Of the DH patient population, 59% self-identified as Hispanic or Latino, 21% needed Spanish medical interpretation, about 57% had Medicaid insurance, and 18% of patients were uninsured. Uninsured patients had access to care via an income-based sliding scale discount program.

Prior to implementation in the CHARM study, DH did not have the infrastructure in place to support the necessary work to identify or manage patients at risk for HCS. There was limited awareness of the importance of the topic and no standard workflow for identification and tracking of at-risk patients. Furthermore, in-house cancer genetic services were not available, so high-risk patients required a referral to outside institutions. These barriers resulted in a limited number of outgoing referrals to cancer-related genetic counseling services.

### DH/CHARM partnership: early involvement of key stakeholders and intentional sustainability focus

To ensure that the CHARM intervention would be feasible to implement and sustain in the DH setting, this study was developed as a collaborative partnership between health services researchers and clinician scientists.

The key personnel at DH included a primary care physician (KA) who led cancer screening initiatives, as well as a medical oncologist (SO) with experience in cancer intervention research trials. Other key DH team members included a patient navigator (EM) and a research coordinator (SD), both of whom identified as Latinx, spoke both English and Spanish, and were experienced in research and clinical care of underserved patient populations. As subject matter experts in the target population and setting, these four DH team members understood the challenges of conducting health services research in a community setting and the clinical and systemic barriers experienced by DH patients. They provided input that would intentionally support not only study design but also sustainability of key study components in future clinical care beyond the study period. They provided input during grant proposal development, assisting in the design of a multimodal study intervention that would act on identified barriers of care for cancer genetic services. The DH team engaged early and directly in the development of the study protocol, the creation of all patient-facing materials, and recruitment and retention methodology.

As part of the study design process, DH study team members also sought input from their clinical colleagues, particularly those in gynecology, family medicine, and oncology, about perceived barriers to genetic testing and counseling in the clinical care setting at DH. The CHARM Study also formed a patient advisory committee (who included but were not limited to PJ and CR) that provided input on the development of study materials and study processes (including patients who preferred Spanish) (Lindberg et al. 2022, O'Daniel et al. 2022).

### Perceived barriers

During the early funding period of CHARM, the DH team readily identified several structural barriers that were limiting successful implementation of the study as well as long-term sustainability efforts. These barriers included:

1. Lack of awareness of HCS: DH clinical colleagues reported limited awareness of HCS beyond their specialty areas, due to lack of formal training or continuing education, and were not comfortable identifying at-risk patients
2. Collection of family history: The US Preventive Services Task Force (USPSTF) (Owens et al. 2019) recommends collection of family history as a first step to identify individuals at risk of HCS. Prior to CHARM, DH had no standard processes or tracked metrics for collecting family history of cancer
3. Documentation of family history: Family history was not consistently documented in the electronic medical record (EMR), as it often competed with more pressing health concerns and was difficult to fit into the limited time allocated to clinic appointments and direct patient care. EMR data pull of family history documentation was measured pre and post intervention
4. Outgoing referrals to cancer genetic services: Family history data needs to be evaluated for HCS risk to determine whether genetic counseling and testing is recommended. DH had only one genetic counselor whose practice was limited to antenatal care; thus, patients considered at-risk based on reported family history or personal history of cancer required a clinician’s referral to genetic counselors at outside institutions. Since these referrals are the last step in the pursuit of genetic testing of at-risk individuals, the number of referrals to genetic counseling services in the EMR was counted pre- and post-intervention as a measure of study impact
5. Documentation of genetic test results: the EMR did not have functionality to easily identify results of genetic testing since these were performed at outside institutions. All results were uploaded into the EMR as generic “outside records” which were difficult to find

## Results

Table 1 summarizes the barriers to HCS testing identified at DH and the solutions and outcomes as a result of the CHARM study.

**Fig. 2:**
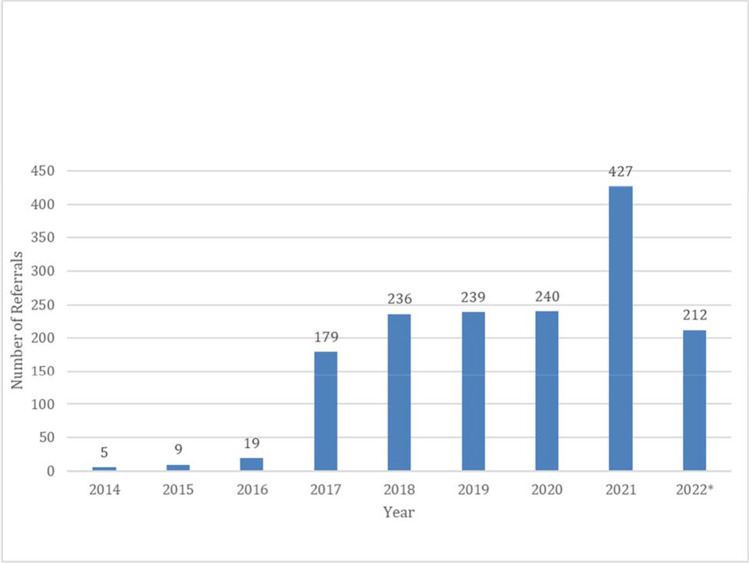
Number of outgoing genetic referrals for HCS at DH. *2022 data is for the first 4 months alone

**Fig. 3:**
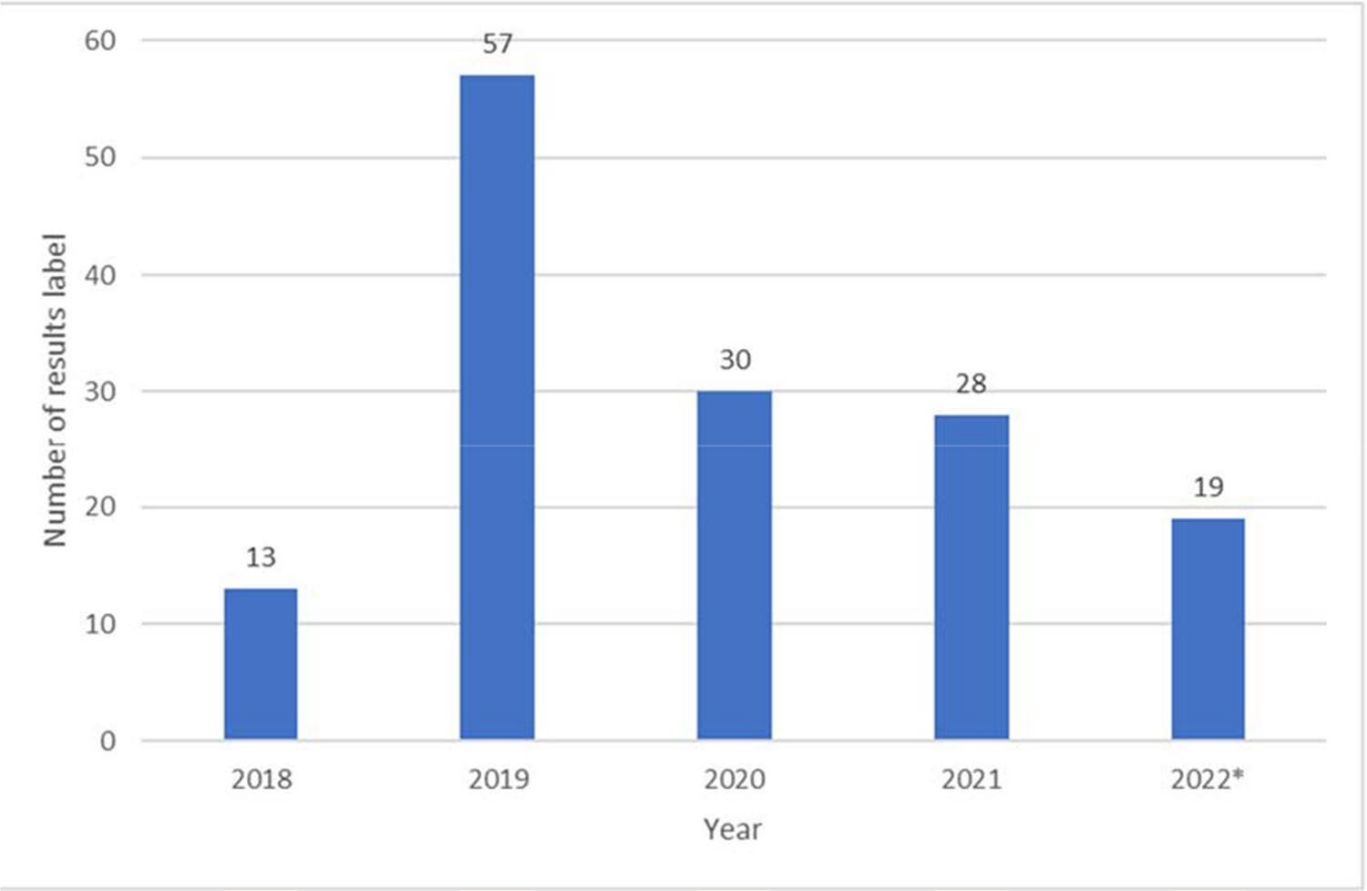
Number of genetic counseling and testing results captured with the “genetic test results” label in the EMR. *2022 data is for the first 4 months alone

1. Lack of awareness of HCS at DH: To overcome this barrier, the DH team used the effort protected by the study’s funding to prepare educational modules on HCS and a list of cancer genetics resources. These modules are lasting resources to be used on an ongoing basis by the DH clinical team and can be easily updated as practice guidelines change. During the study, the HCS educational modules were presented to five primary care clinics and other provider groups attuned to HCS and genomics (women’s care, gastroenterology, and oncology). A total of 14 educational sessions were held, across nine clinical sites and four departmental meetings within DH, with a total attendance of 161 clinicians and 219 key stakeholders, which included patient navigators in the gastrointestinal (GI) lab, nursing, and cancer work groups.
2. Collection of family history: To support the study protocol, the team developed a patient-facing, electronic family history collection and risk assessment application that was accessible independent of the clinical visit or involvement of the clinician (Mittendorf et al. 2022b, Mittendorf et al. 2022a). During the study period, 364 DH patients successfully completed the family history collection tool that was developed for CHARM.
3. Documentation of family history: To create a sustainable change around family history collection, DH developed a standard workflow for documenting family history in the EMR, with ongoing work exploring the implementation of family history collection tools in key clinical areas, including the mammography and endoscopy units. This has resulted in an increase from 65% pre-intervention to 70% post-intervention of family history documentation in the EMR of patients who receive primary care services in the first 18 months of implementation.
4. Outgoing referrals to cancer genetic services: Prior to CHARM, few referrals per year were placed for patients at risk for HCS, and most of these were for patients who already had a diagnosis of cancer. The CHARM family history tool (Mittendorf et al. 2021) streamlined this process, eliminating the need for a genetic counselor to review the family history data. Genetic testing and counseling were offered within the study without the need for a clinic visit or outside referral. After the study, with the increased awareness of HCS, resource compilation, and implementation of standardized workflows for the components above, DH saw a marked increase in referrals to genetics for the indication of evaluation of HCS during CHARM which persisted post-intervention (Fig. 2). In 2021, DH placed 427 referrals to genetics for this indication alone compared to 240 in 2020. In 2022, 212 referrals were placed in the first 4 months alone, suggesting that this change will be sustained.
5. Documentation of genetic test results: The DH team created a specific bar code label and process to clearly identify genetic test results in the EMR. “Genetic results” identified by the newly created label increased during study enrollment (2019–2020) due to the number of participants receiving genetic results as part of the study and continues to be used at a lower steady-state level in 2021 and 2022, after study completion, as part of ongoing regular clinical care (Fig. 3).

**Table 1:** Summary of identified barriers and outcomes after the CHARM study intervention

Barrier	Before CHARM	During CHARM	Outcomes because of CHARM
Lack of awareness of HCS	Limited awareness and engagement surrounding HCS and cancer genetic services	Early stakeholder engagement, design focused on sustainability, creation of educational modules	Educational modules shared with 161 clinicians and 219 key stakeholders and available for future use
Collection of family history	No standard workflow to collect family history	Development of risk assessment tool	364 DH patients successfully completed the risk assessment tool
Documentation of family history	No standard workflow to document family history	Development of standard workflow to document family history in the EMR	Family history documentation increased from 65% pre-intervention to 70% post-intervention
Outgoing referrals to cancer genetic services	Number of referrals to cancer genetic services was 179 in 2017 (year prior to intervention)		Consistent increase in number of referrals, up to 427 in 2021 (year after CHARM study was completed)
Documentation of genetic test results	No standard workflow to document genetic results in chart	Creation of genetic test results label in EMR	Genetic results label in the EMR continues to be used

## Discussion

DH is a safety net institution and prior to the CHARM Study had no formal infrastructure in place to offer hereditary cancer risk assessment or cancer genetic services. By participating in CHARM, DH created a lasting roadmap for the ongoing development of such services, while making key contributions to the success of the research project. Instrumental to the success of and post-study sustainability of the CHARM intervention was the early involvement of key stakeholders, including clinical leaders, a variety of providers, and a patient advisory committee. While early engagement of community partners is an essential feature of sustainable translational research (Barger et al. 2019, Forsythe et al. 2016, LeClair et al. 2020), it is critical to choose the right partners who have leadership buy-in for long-term sustainability. Another characteristic of this partnership was the intentional focus on developing and creating a sustained improvement in the identification and care for hereditary cancer patients for our institution, adopted by all partners in the project, starting long before the first participant was enrolled.

Once key coinvestigators and team personnel were identified, the process started by raising awareness of the importance of HCS via educational modules delivered to 161 clinicians and 219 key stakeholders throughout the institution. The DH team then established best practices and standardized workflows for the collection of family history, documentation of high-risk patients within the problem list, and creation of a specific label in the EMR to flag genetic testing results for easy identification. While these were implemented as part of the study protocol, because of the sustainability of the intervention design, these workflows continue to be used clinically after study completion.

Due to increased awareness and workflows created as part of the study, DH has seen a substantial and sustained increase in the number of referrals to genetics for HCS, from less than 100 referrals pre-intervention to over 400 referrals a year post-intervention, reflecting a considerable positive impact in the health system, paving the way to identifying more patients at risk for HCS. It is worth noting that the CHARM study offered free genetic testing and posttest genetic counseling to participants, which theoretically should have decreased the number of genetic counseling referrals post-intervention. The fact that it did not, despite the recruitment success of the study, speaks to the larger impact of the study in the institution, likely grounded on the increased awareness of the topic of HCS for the entire healthcare system. We observed only a modest increase in family history documentation in comparison to the increase in genetics referrals, suggesting that the strongest contributor of the study’s lasting impact was the increased awareness and understanding of HCS. This demonstrates that it is critical that clinicians understand *why* collecting this information is important in order to *act* upon this information by recommending further evaluation via a referral.

A remaining challenge is the actual completion of genetic counseling and testing when this resource requires a referral to an outside institution unfamiliar to the patient. Anecdotally, DH clinicians report that many patients do not end up setting up appointments and/or getting tested. To eliminate the need for outside referrals, DH is working toward expansion of in-house genetic counseling services to include HCS. This will increase timely access to genetic services and improve care access and coordination for DH patients.

Participating in the CHARM study has led to several important and sustainable changes at DH. DH providers have recognized that risk assessments for HCS can be completed without interrupting clinical workflows in a busy, under-resourced primary care setting by using patient-facing or staff-directed screening tools for HCS outside of the clinical encounter with a provider. Currently, the DH team is exploring implementing risk assessment tools in the mobile mammography van to increase access in other underserved areas. Finally, DH is establishing a prospective HCS registry, with the long-term goal of setting up a medical home with an established care team for these patients. The team will be using a model championed by Kaiser Permanente Colorado, which leverages the EMR and allows for tracking of HCS patients.

## Conclusion

The CHARM study is an example of a mutually beneficial academic-community partnership. Our approach outlines a template for researchers and clinical leaders seeking to design sustainable interventions to improve access to cancer genetic services in a safety net setting for underserved populations. Key to its success was the early involvement of the appropriate clinical leadership and clinician stakeholders, patient engagement in the research process, and dedicated and intentional efforts to make the research venture endure past the funding period by focusing on infrastructure building and knowledge transfer.

## Data Availability

Data: Not applicable Materials: study materials available at https://cser-consortium.org/
